# Comprehensive Evaluation of Uropathogens’ AMR in a Romanian Tertiary Center: Male vs. Female Comparison

**DOI:** 10.3390/microorganisms14061236

**Published:** 2026-05-30

**Authors:** Răzvan-Ionuț Popescu, Cristian Toma, Răzvan-Cosmin Petca, Cristian Mareș, Leonard Ostafi, Aida Petca, Viorel Jinga

**Affiliations:** 1 Faculty of Medicine, “Carol Davila” University of Medicine and Pharmacy, 8 Eroii Sanitari Blvd., 050474 Bucharest, Romania; dr.razvanp@gmail.com (R.-I.P.); cristian.toma@umfcd.ro (C.T.); dr.marescristian@gmail.com (C.M.); leonardostafi@gmail.com (L.O.); vioreljinga@yahoo.com (V.J.); 2 Department of Urology, “Prof. Dr. Theodor Burghele” Clinical Hospital, 20 Panduri Str., 050659 Bucharest, Romania; 3 Department of Urology, “Saint John” Clinical Emergency Hospital, 13 Vitan-Barzesti Str., 042122 Bucharest, Romania; 4 Department of Obstetrics and Gynecology, CF2 Clinical Hospital, 63 Marasti Blvd., 011464 Bucharest, Romania

**Keywords:** uropathogens, AMR, Gram-negative, *E. coli*, fluoroquinolones

## Abstract

Introduction: Urinary tract infections (UTIs) represent a growing concern in both clinical practice and public health, affecting hospitalized and outpatient populations across all ages and genders. This study aims to evaluate the prevalence of uropathogens and their antimicrobial resistance profiles in male and female patients comparatively at a tertiary urological center. Materials and Methods: A retrospective descriptive analysis was conducted, covering three identical 6-month periods—September 1 to February 28—in three consecutive years from 2023 to 2025. The study included 2270 male patients (2270. 57.06%) and 1708 female patients (1708. 42.94%), all with at least one positive urine culture (>10^5^ CFU/mL). Data on age, gender, bacterial species, and antimicrobial agents were collected and analyzed. Results: A higher prevalence of Gram-negative bacteria was observed compared to Gram-positive bacteria in both male (1752; 77.18% vs. 518; 22.82%) and female (1369; 80.15% vs. 339; 19.85%) groups. The most common microorganisms were *Escherichia coli*, followed by *Klebsiella* and *Enterococcus*. *Klebsiella* showed high rates of antimicrobial resistance, especially in males, across various antibiotic classes such as amoxicillin-clavulanic acid (60.6% vs. 43.25%), levofloxacin (40.18% vs. 27.91%), aztreonam (37.4% vs. 27.27%), and ceftazidime (36.23% vs. 24.03%). High resistance levels, although not statistically significant, were also noted for trimethoprim/sulfamethoxazole (43.64%) and nitrofurantoin (65.69%). In males, *E. coli* exhibited higher resistance rates to trimethoprim/sulfamethoxazole (44.65% vs. 32.89%), levofloxacin (43.27% vs. 30.78%), and amoxicillin-clavulanic acid (40.18% vs. 27.19%). Carbapenems remained highly susceptible in both groups. *Enterococcus* showed similar resistance patterns in both cohorts, primarily resistant to penicillin and levofloxacin. Conclusion: This study highlights higher resistance rates among Gram-negative bacteria in males to commonly used antibiotics such as fluoroquinolones, trimethoprim/sulfamethoxazole, and β-lactams. Resistance patterns in Gram-positive bacteria remained stable across both populations, with high susceptibility to fosfomycin, nitrofurantoin, linezolid, and carbapenems. Differences between sexes emphasize the need for more detailed analysis of local and sex-specific resistance patterns.

## 1. Introduction

Urinary tract infections (UTIs) represent a raising concern of both clinical practice and public health, affecting both male and female patients, regardless of age. Fine assessment of UTI etiology and management is even more important, given that they represent one of the most important causes of morbidity and antibiotic therapy for over 150 million individuals each year, not only in hospitals but also in the general population [1].

A systematic analysis by Zeng Z. et al. showed that the high incidence of UTIs is observed in the aging population, with increased life expectancy, greater exposure to various medical and surgical interventions, and a high prevalence of comorbidities [2]. The socioeconomic impact due to increased medical costs, multiple hospital admissions, diagnostic procedures, and the decrease in productivity of patients is a fundamental aspect described and analyzed in the last decade [3]. Recurrence of the disease also negatively impacts the quality of life [4].

A major challenge of the current healthcare system is the significant increase in antimicrobial resistance (AMR) rates, resulting in high treatment failure rates under empirical antibiotic therapy [5]. According to the surveillance studies performed by Pitout JDD et al., *Escherichia coli* and *Klebsiella* spp., two of the most commonly encountered uropathogens, exhibit some of the most complex antimicrobial resistance patterns [6]. To achieve maximum efficiency in patients receiving empiric antibiotic therapy and to prevent the growing prevalence of multidrug-resistant bacteria, it is essential to monitor the prevalence and distribution of resistant microorganisms and the rates of antimicrobial resistance through similar surveillance studies [7].

In response, the World Health Organization (WHO) developed a series of coordinated initiatives to improve surveillance systems and promote antimicrobial stewardship strategies [5]. The Global Surveillance System for Antimicrobial Resistance and Antimicrobial Use (GLASS) was implemented in 2015, with the primary aim of standardizing the collection and reporting of AMR data globally. The constant and continuous monitoring of resistance trends to various pathogens using standard biological samples, such as urine culture, illustrates how this program operates [8,9,10]. Romania provides data on AMR trends continuously through programs supported by both the European Union (EU) and the WHO, such as EARS-Net and CAESAR. Analyzing reported data on Eastern Europe, we understand that the most commonly encountered bacteria, especially Gram-negative microorganisms, exhibit high rates of AMR, and thus both the prevalence of various species and the associated levels of resistance represent appropriate objects of study [11].

In the specific context of UTIs, gender and age also play an essential role. The anatomical differences between male and female patients (shorter urethral length and the increased risk of colonization due to proximity to the anorectal area in the latter) translate into different prevalences between genders. It is of great importance to mention that female patients exhibit predominantly uncomplicated urinary tract infections and present two peaks of incidence (the sexually active period and the postmenopausal period) [1]. Considering male anatomy as a protective factor against infection, the prevalence is lower, and the nature of UTI is characterized as complicated. In most cases, a structural abnormality of the urinary tract, an obstructive pathology such as bladder outlet obstruction (BOO), urolithiasis, or intermittent/indwelling catheterization, is involved [12].

Some of these conditions are strongly correlated with age, especially benign prostatic hyperplasia (BPH), a common pathology that mainly affects male patients after the age of 50–60 years, and thus the prevalence of UTIs is higher in this age group [13]. Other factors related to age consist of exposure to various medical services, urinary catheterization or urinary tract instrumentation, and the presence of chronic comorbidities [14]. Analyzing data separately for male and female populations allows assessment of differences in the prevalence and distribution of involved microorganisms, enabling more accurate conclusions regarding predisposing factors. Given the higher incidence of UTIs, treatment guidelines are most often based on prevalence and resistance data in female populations [15,16]. The need for specific research is underscored by recent studies suggesting marked differences in prevalence and resistance profiles between male and female populations [17].

Despite the extensive literature on UTI epidemiology and antimicrobial resistance, important gaps remain. Most available data and clinical guidelines are derived from female or mixed populations, while male UTIs, which are more often complex infections, are underrepresented [1,18]. In addition, there is a lack of contemporary data about the post-COVID period, during which antibiotic prescriptions have significantly increased. For the Eastern European countries, despite continuously increasing antibiotic resistance rates, the local reported data remain insufficiently reported by the surveillance systems [10,19].

This study aims to compare the distribution of local UTI prevalence and antimicrobial resistance trends among male and female patients selected at 6-month intervals over 3 years. Given the large cohorts, we aim to provide current and realistic epidemiological data regarding the Romanian population. The primary objective is to compare the two study groups with respect to age and bacterial prevalence. The secondary objective is to evaluate antimicrobial resistance profiles across different pathogens and identify statistically significant differences between the two groups. By addressing these gaps, this study aims to generate clinically relevant data that may support more accurate and tailored empirical antimicrobial therapy in both male and female populations.

## 2. Materials and Methods

We conducted a retrospective descriptive analysis comparing data on the prevalence of different uropathogens and, subsequently, the associated antimicrobial resistance profiles at a tertiary urological center and a major academic center in Romania—the “Prof. Dr. Th. Burghele” Clinical Hospital.

The study included three similar 6-month observation periods between the 1st of September and the 28th of February, in three consecutive years, from 2023 to 2025, selecting 2 different seasons (autumn and winter) that correlate with high presentation rates and patient admissions in our hospital. The same periods were successfully used in previous studies regarding the same topic conducted in our department [11,18].

**Figure 1:**
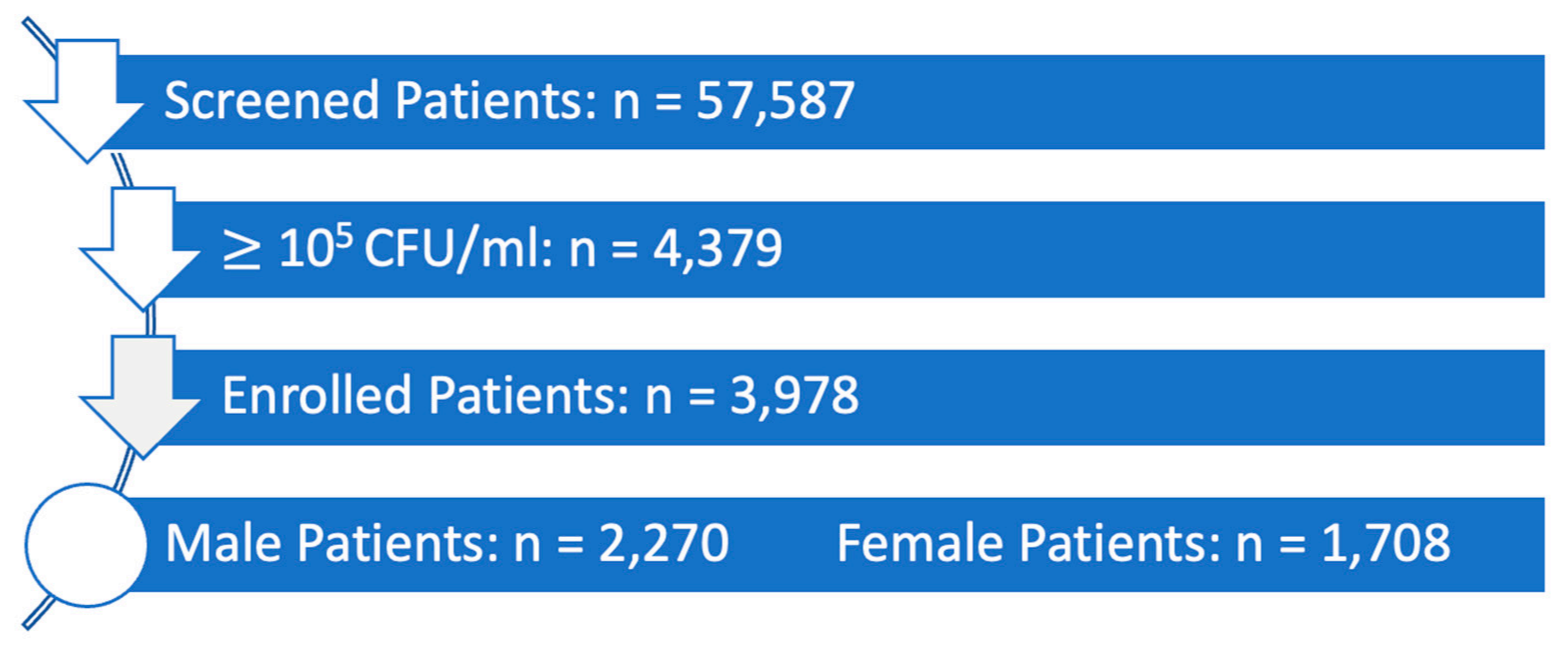
Graphical representation of patients assessed and included in the study group.

We included both male and female patients with a positive urine culture during the assessed period (see Figure 1 for patients’ selection). To avoid data contamination, only cultures recorded in the hospital’s microbiology laboratory were considered valid. Given that included patients were treated both as outpatients and as inpatients, an analysis of the most common types of pathologies recorded and their correlations with prevalence and resistance data, as well as a classification into single or recurrent episodes of infection, could not be included in the study. In contrast to these limitations, a degree of uniformity in the distribution is provided.

Cases with similar urine culture results for the same patient at two successive evaluations were excluded to prevent duplicate results. Also, when multiple urine cultures were identified for the same patient, only the first positive culture was recorded. The identification was made based on the personal identification number existing in the medical files.

The Prof. Dr. Theodor Burghele Clinical Hospital is among the referral centers and serves an area comprising approximately 30% of the country’s total population, receiving some of the most challenging cases referred from collaborating centers. It has over 135 beds and treats a wide range of conditions, from renal-ureteral lithiasis to benign, functional, and urological pathologies. Given these factors, the data analyzed in this study can provide robust information based on a substantial population with a varied regional distribution.

The study adhered to the criteria set forth by the Declaration of Helsinki for research involving human subjects. Additionally, the hospital’s Ethics Committee approved the study methodology, which also guarantees the confidentiality of patient data. Because of the study’s retrospective design, patient consent was not required for inclusion in the database. However, the study was presented to the patients, and consent was obtained at the time of admission, given that this is an academic hospital where clinical studies are conducted, and the consent form includes legal provisions that patients can accept.

The inclusion criteria consisted of a positive urine culture, defined as the presence of at least 10^5^ CFU/mL, a single bacterial species identified on culture, and the age of at least 18 years old. The cases with bacteriuria < 10^5^ CFU/mL, multiple bacterial species identified, asymptomatic bacteriuria, patients with urinary catheters, and lithangiuria were excluded from the study. Duplicate urine culture results from the same patient were excluded (based on the personal identification number, only the first positive urine culture was registered).

The European Association of Urology (EAU) updated guidelines on Urological Infections served as the primary source of guidance in the context of empiric antibiotic therapy. Several agents were selected based on validated antibiograms and local prevalence data previously described in studies conducted on Romanian populations, which represented an adjuvant in choosing the most appropriate antimicrobial therapy [14]. Whenever necessary, an interdisciplinary consultation between the Departments of Urology and Infectious Diseases was performed.

For the microbiological evaluation of the samples obtained, laboratory protocols were followed, including culture, identification of microorganisms, and susceptibility testing against a wide range of antibiotics.

All of these protocols, including the Kirby–Bauer method for determining susceptibility, have been described in numerous previous studies conducted at the same hospital [20].

All analyzed data were collected using Microsoft Excel 16.0 (Microsoft Corporation, Redmond, WA, USA; version 16.0). Descriptive statistical analyses were performed in Python version 3.8.9 (Python Software Foundation, Wilmington, DE, USA), using JupyterLab 3.2.4 (Project Jupyter; https://jupyter.org/) and the Pandas library 1.3.4 (pandas development team; https://pandas.pydata.org/). Categorical variables were expressed as absolute frequencies and percentages. Data comparison between groups regarding antimicrobial resistance rates was assessed with the chi-square test, and when expected cell counts were under 5, Fisher’s test was applied. Statistical tests were considered significant at a *p*-value < 0.05. Grammarly (https://www.grammarly.com), and DeepL (https://www.deepl.com/en/translator (accessed on 23 March 2026)) were used for language accuracy as none of the authors is a native English speaker.

## 3. Results

This study included 3978 patients with positive urine cultures (Figure 1). Of these, 2270 were men (57.1%) and 1708 were women (42.9%). Patients over 55 years of age represented 79.3% of the total patients included in the study, and 20.7% were under 55 years of age (Table 1). Among men, 89.11% were over 55 years of age, and among women, the percentage was significantly lower, at 66.33%. Observable differences were also observed in the average age of patients: 70.34 years among male patients and 59.81 years among female patients. These data reveal an increased prevalence of older men at the time of diagnosis.

As can be observed in Table 2, the patients’ settings slightly differed between inpatient and outpatient settings in both sexes. Around 60% of the patients were registered as an ambulatory setting, mostly due to the fact that pre- and postoperative evaluations are made as short visits to the hospital unit.

Following the microbiological analysis performed after sample processing, differences were observed in the distribution and prevalence of the identified bacterial agents. *Escherichia coli* was the predominant etiological agent in both groups. Among female patients, it presented a higher prevalence of 54.1%, compared to 32.42% in men. Among male patients, *Klebsiella* spp. was diagnosed more frequently (25.81% vs. 15.4% in women). The observed variations in prevalence by patient sex are likely influenced by the characteristics of the underlying pathology. Unlike Gram-negative species, where there were important sex differences, *Enterococcus* spp. was identified in comparable proportions across the two groups (15.63% in women vs. 18.68% in men). The prevalence data expressed as percentages or numerical data are presented in Table 3 and Table 4.

The distribution of the different bacterial species did not significantly differ between inpatient and outpatient subgroups in either sex. Statistical analysis demonstrated no significant variation in microbiological profiles between the two cohorts, suggesting a relatively homogeneous bacterial spectrum regardless of hospitalization status (Table 5). The absence of statistically significant differences in baseline bacterial distribution indicates that the microbiological characteristics of the two subgroups were comparable.

The evaluation of antimicrobial resistance profiles revealed differences in both Gram-negative and Gram-positive bacteria, depending on patients’ gender (Table 6 and Table 7).

For Gram-negative bacteria, higher rates were observed for several antibiotic classes, including levofloxacin (42.96% vs. 31.48%), trimethoprim/sulfamethoxazole (46.38% vs. 34.64%), and amoxicillin-clavulanic acid (49.14% vs. 30.92%) (Table 4). A similar profile was also observed for the broad-spectrum antibiotics aztreonam, ceftazidime, or carbapenems.

Gram-positive bacteria did not show significant differences in distribution by gender, but they exhibited very high resistance rates to some antibiotics (Table 7).

High rates of antimicrobial resistance were observed with fluoroquinolones, such as levofloxacin (49.89% in men and 39.02% in women), and penicillin (42.49% in men and 38.32% in women).

The highest rates of resistance identified were observed for fluoroquinolones, such as levofloxacin (49.89% in men vs. 39.02% in women), and penicillin (42.49% in men vs. 38.32%). The results favorably highlighted the maintained efficacy of antibiotics such as linezolid, fosfomycin, vancomycin, and nitrofurantoin against Gram-positive bacteria in both groups.

The results highlight differences in resistance rates, more pronounced for Gram-negative bacteria, while Gram-positive bacteria remain sensitive to multiple classes of antibiotics.

Regarding the sensitivity to various classes of antibiotics, *Escherichia coli* showed important differences between the two sexes (Table 8). As the predominant etiological agent in both populations, it showed higher resistance to trimethoprim/sulfamethoxazole (44.65% vs. 32.89%) among male patients. Also, higher rates of resistance were identified among men for levofloxacin (43.27% vs. 30.78%) and amoxicillin–clavulanic acid (40.18% vs. 27.19%). There are notable differences in resistance rates, with higher rates in men, even for antibiotics for which the overall resistance level remains below 20%, such as aztreonam, ceftazidime, fosfomycin, and nitrofurantoin. Regarding effective therapeutic options, with increased sensitivity and viability in clinical practice for male patients, amikacin and carbapenems are valid options. Although fosfomycin and nitrofurantoin have sensitivity rates over 91%, their use is limited in men, with these agents mainly recommended for the treatment of uncomplicated urinary tract infections.

For *Klebsiella*, the second most common uropathogen in men, a distinct resistance profile was evident within the studied cohort (Table 9). Significantly higher rates of antimicrobial resistance were observed for amoxicillin-clavulanic acid (60.6% vs. 43.25%), levofloxacin (40.18% vs. 27.91%), aztreonam (37.4% vs. 27.27%), and ceftazidime (36.23% vs. 24.03%). Additionally, although not statistically significant, high levels of resistance were observed for trimethoprim/sulfamethoxazole (43.64%) and nitrofurantoin (65.69%). Another relevant aspect, supported by statistically significant differences, is resistance to carbapenems, which, among the male population, is 23% for imipenem and 18.18% for meropenem.

Data from this study reveal significantly higher levels of antimicrobial resistance among men for most antibiotics studied for *Klebsiella*, compared to the female population.

In the case of *Pseudomonas*, unlike the resistance profiles described for *Escherichia coli* and *Klebsiella*, no significant differences were observed between the two groups analysed, and antimicrobial resistance was maintained at high values for both sexes (Table 10). Thus, for frequently used antibiotics, the level of resistance was high: 49.53% for levofloxacin, and 30.84% for amikacin. A notable feature is the high level of resistance to last-line antibiotics such as aztreonam and carbapenems. In the male population, resistance was 44.24% for aztreonam, 34.93% for meropenem, and 38.83% for imipenem.

The study data indicate similar resistance values in men and women, with high resistance across multiple antibiotic classes.

In the case of *Proteus*, the trend of high antimicrobial resistance rates persists, especially among male patients, across several antibiotic classes (Table 11). The highest resistance was evidenced for amoxicillin-clavulanic acid (47.435% in men vs. 29.09% in women). High resistance rates were recorded for trimethoprim/sulfamethoxazole (61.61%) and levofloxacin (42.63%), but without any statistical difference. Similarly, *Proteus* exhibits increased resistance to imipenem.

*Enterococcus* showed a similar antibiotic resistance profile, with the highest rates observed for levofloxacin, and penicillin (Table 12). The values exceeded 30% for penicillin and 48.83% for levofloxacin among the male population.

Antibiotics such as fosfomycin, linezolid, nitrofurantoin, and vancomycin maintained high sensitivity.

With regard to *Staphylococcus*, unlike other Gram-positive cocci, this bacterial species showed statistically significant differences in resistance to certain classes of antibiotics among the studied populations. Table 13 shows a significantly higher resistance rate in the male population regarding ampicillin (81.81% vs. 55.88%) and levofloxacin (54.65% vs. 24.24%). Sensitivity rates above 90% were observed for nitrofurantoin, linezolid, and carbapenems in both cohorts.

The analysis of the susceptibility of Gram-negative and Gram-positive bacteria in this study, in relation to the sex of the patients, highlighted significant differences for several classes of antibiotics, but also common patterns in evolution, observed depending on the type of bacterial agent analyzed. For Gram-negative bacteria, differences in resistance rates are observed for amoxicillin-clavulanic acid, trimethoprim/sulfamethoxazole, and fluoroquinolones. The most pronounced differences are observed in *Escherichia coli*, *Klebsiella*, and *Proteus*. Regarding *Pseudomonas*, although resistance was high, there were no statistical differences between the sexes.

Gram-positive bacteria show similar trends in resistance and susceptibility across the two groups. They exhibited high resistance to ampicillin and levofloxacin, while sensitivity remained constant for fosfomycin, nitrofurantoin, linezolid, and vancomycin.

## 4. Discussion

### 4.1. Epidemiology and Demographic Data

Our study’s analysis of demographic data and bacterial prevalence is similar to and consistent with the trends observed in other recent epidemiological studies on UTIs. The data obtained and aligned to the recent literature confirm the positive aging pattern of the human population and a positive increase in the prevalence of urological pathology. This situation leads, proportionally, to an increase in both the prevalence and incidence of UTIs, with direct implications for the elderly population [1,5]. In addition, recent studies suggest that the elderly are more likely to develop infections caused by opportunistic pathogens, including Gram-positive organisms such as *Enterococcus* spp., which are frequently associated with healthcare-related infections and prior antibiotic exposure [21]. According to recent studies, 55 years old represents a landmark for the male population to develop urinary tract infections related to underlying obstructive pathologies [22], in comparison to females, who present a different distribution during their lifetime. The present results also reveal that the vast majority of UTIs were diagnosed in this age group.

*Escherichia coli* remains the leading pathogen among male patients, though at significantly lower rates (32.42%) than among female patients (54.1%). These data correlate with findings from globally assessed populations in specialized studies focused on female subjects, which report a prevalence of 60% to 80% [4,17]. An increase in the *Klebsiella* prevalence pattern was also observed, the second most common isolated bacteria. The prevalence rate among males was 25.81%. This may reflect the higher frequency of complicated infections, prior instrumentation, and healthcare exposure in this group [4,23]. The shift in pathogen distribution highlights the distinct microbiological profile of male UTIs and supports the need for sex-specific epidemiological assessment in clinical practice. This prevalence exceeds the rates reported in other epidemiological findings [4].

When evaluating the bacterial prevalence among inpatients and outpatients in both sexes, there were no statistically significant differences. This observation may be explained by the fact that, although classified as outpatients according to the timing of urine culture collection, many of these patients had substantial prior or subsequent contact with the hospital environment, including surgical interventions performed before or after sample collection. From this perspective, the outpatient cohort did not represent a truly ambulatory-managed population, but rather a group with a history of relevant hospital admissions. For this reason, we did not aim to make a comparison between these two subgroups, but rather an evaluation of different prevalence rates of antimicrobial resistance patterns among the main microorganisms involved in UTIs development.

### 4.2. Overall Bacterial Resistance Comparison

When discussing the overall resistance pattern identified for both Gram-negative and Gram-positive microorganisms across both sexes, the most pronounced differences and increasing trends in antibiotic resistance were observed in male patients. This pattern was confirmed by other recent studies. The male study group exhibited higher resistance rates, especially to levofloxacin (42.96% vs. 31.48%), amoxicillin-clavulanic acid (49.14% vs. 30.92%), and trimethoprim/sulfamethoxazole (46.38% vs. 34. 63%). Nowadays, the literature analysing resistance rates in UTIs generated by *Escherichia coli* demonstrates local differences ranging from 30 to 40% for trimethoprim/sulfamethoxazole and from 25 to 35% for fluoroquinolones [24,25]. Sensitivity rates, although slightly positive, increased and remained within acceptable limits for antibiotics such as carbapenems (imipenem, 15.79% in men vs. 3.34% in women) and aminoglycosides (amikacin, 13.14% in men vs. 9.31% in women). The information obtained is consistent with recently published reports demonstrating that both classes, aminoglycosides and carbapenems, remain viable treatment options for Gram-negative uropathogens, at least for the moment [26].

In our study, the general resistance rates of Gram-positive bacteria demonstrated similarities between both sexes. The highest resistance rates identified were for fluoroquinolones such as levofloxacin (49.89%), a class that is highly restricted and subject to intense antibacterial stewardship worldwide. Relatively close to levofloxacin resistance rates are penicillin (42.49%) and amoxicillin-clavulanic acid (34.62%). Antibiotic susceptibility patterns with values above 90% were observed for several antibiotics, including nitrofurantoin, fosfomycin, linezolid, and vancomycin. According to the current databases, recent studies demonstrate that for Gram-positive infections, particularly those caused by *Enterococcus*, glycopeptides and oxazolidines remain viable treatment options [23].

In this study, we observe that while Gram-positive bacteria exhibit more stable resistance patterns, with sustained susceptibility profiles for a wide range of antibiotics, the other type of Gram-negative microorganisms exhibit the highest rates of antimicrobial resistance, making it an increasingly significant challenge for clinical practice and adherence to treatment guidelines [14,16].

Despite general agreement with previously published epidemiological studies, the prevalence of antimicrobial resistance observed in the present study, particularly among male patients, appears to be higher than reported in predominantly community-based or mixed cohorts. Large surveillance studies have reported lower resistance rates compared to our findings [25,26,27]. This differences may be explained by the tertiary-care setting, where patients frequently present with prior antibiotic exposure, repeated hospitalizations, and complex urological conditions. All of these factors are established drivers of antimicrobial resistance [4,28,29].

Sex-specific differences in antimicrobial resistance remain insufficiently explored in the literature. Male UTIs are more frequently classified as complicated infections and are usually associated with structural abnormalities such as bladder outlet obstruction, urolithiasis, or prostatic involvement [12,14,17]. These factors often require repeated antibiotic therapy and urological interventions, thereby increasing selective pressure and promoting the emergence of multidrug-resistant organisms. Our findings add to the existing literature by demonstrating that male patients constitute a distinct subgroup with a higher rate of antimicrobial resistance.

### 4.3. Gram-Negative Comparison

Significant variations in susceptibility and resistance to various general classes of antibiotics were demonstrated by the analysis of distribution by bacterial species among Gram-negative microorganisms in the study population.

With the highest prevalence rate even among the male population, *E. coli* demonstrated significantly different resistance rates for amoxicillin-clavulanic acid (40.18% vs. 27.19%), levofloxacin (43.27% vs. 30.78%) and trimethoprim-sulfamethoxazole (44.65% vs. 32.89%)- with the last antibiotic resistance being the highest. In accordance with our findings, the recent literature on *E. coli*-related UTIs demonstrates increased resistance rates to commonly used clinical practice antibiotics such as fluoroquinolones and trimethoprim/sulfamethoxazole [30,31].

Variations in antibiotic resistance rates are observed from children to adult but it also appear to be closely related to the elderly population and to high prior antibiotic treatment in male patients for the treatment of recurrent or complicated UTIs [26,31]. In *Escherichia coli*’s case, it was found in this study that a number of antibiotics maintained a stable sensitivity rate, at least for amikacin and carbapenems, for both patient groups (>90%). In addition, a very good sensitivity rate was also obtained for fosfomycin and nitrofurantoin, supporting the utility of these drug classes for the treatment of urinary tract infections- one may say a re-emergent treatment strategy. Although we should highlight that their use is more appropriate for female patients with uncomplicated urinary tract infections, such as cystitis, where they remain a therapeutic option of choice [26,32,33].

*Klebsiella* isolates showed statistically significant differences in gender-related bacterial resistance rates for a vast range of the studied antibiotic treatments. For treatment options such as amoxicillin-clavulanic acid (60.6% vs. 43.25%), trimethoprim/sulfamethoxazole (43.64% vs. 31.08%), aztreonam (37.4% vs. 27.27%), ceftazidime (36.23% vs. 24.03%) or levofloxacin (40.18% vs. 27.91%), alarming data were demonstrated. This analysis confirms the evolution of similar patterns previously identified in other specialty studies, which show increased rates of *Klebsiella* resistance to β-lactams and fluoroquinolones [34]. An increased prevalence of complicated infections among men is directly associated with higher rates of resistance in male patients [35]. Notable data regarding this pathogen were also identified in males concerning resistance to both imipenem (23.1%) and meropenem (18.18%). These data confirm international statistics describing a significant increase in multidrug-resistant *Klebsiella* isolates in urinary tract infections [36].

The particularly high resistance rates observed in *Klebsiella* spp., including carbapenem resistance found in this study appear to be higher than global epidemiological trends [6,35,37,38,39]. This finding may reflect cumulative healthcare-associated exposure, as *Klebsiella* infections are strongly linked to nosocomial settings, invasive procedures, and prior use of broad-spectrum antibiotics [29,35]. Since our patients are mostly elderly, this may be an explanation for the reason why resistance to *Klebsiella* registered such increased values. In addition, the elevated resistance to nitrofurantoin observed in our study is consistent with the limited activity of this agent against this species, further restricting therapeutic options [34].

From a mechanistic perspective, the increased antimicrobial resistance observed in male patients may be explained by chronic urinary retention, prostatic involvement, and biofilm formation, which facilitate bacterial persistence and reduce antibiotic penetration [12,39]. Biofilm-associated infections, particularly in the context of urolithiasis or urinary tract instrumentation, decrease antimicrobial efficacy and lead to resistance development [40,41]. These mechanisms may partly explain the higher resistance rates observed in our cohort.

Even though, in previously discussed studies, *Pseudomonas* showed no significant differences in reported distribution patterns for antibiotic resistance rates, in our study, higher rates were identified for most of the antibiotics studied, similar to trends observed for *E. coli* and *Klebsiella*. These data may indicate that the level of resistance in *Pseudomonas* is not influenced by the patient’s sex, but it is rather directly associated with the bacteria’s own intrinsic resistance mechanisms or those associated with the healthcare context. This pathogen exhibits multiple adaptive strategies, including efflux pumps, reduced outer membrane permeability, and rapid acquisition of resistance determinants under antibiotic pressure [42,43]. Nowadays, data indicate that this bacterial species often exhibits increased resistance rates to various classes of antibiotics such as carbapenems, cephalosporins, and fluoroquinolones [1,4]. Multidrug-resistant *Pseudomonas* has been frequently associated in clinical studies with healthcare settings (the quality and rigidity of local health services), the presence of urinary catheters, and institutionalization [1,42]. The high resistance rates observed in both sexes are likely driven by bacterial factors rather than host-specific characteristics. Nevertheless, the elevated resistance to carbapenems and fluoroquinolones is clinically concerning and aligns with global data identifying *Pseudomonas aeruginosa* as a major multidrug-resistant pathogen in healthcare-associated infections [43,44].

Correlation of these data might imply that bacterial resistance in *Pseudomonas* species is primarily due to intrinsic mechanisms, rather than to healthcare settings, and is not associated with patient gender distribution.

Similar results were obtained for *Proteus* bacteria. *Proteus* isolates in male patients exhibited higher resistance rates more frequently than in women with *Proteus*. Statistically significant increases in resistance were observed for imipenem, amoxicillin-clavulanic acid, and ceftazidime. In our study, similar data showed increased resistance rates for ampicillin, trimethoprim/sulfamethoxazole, and levofloxacin. According to recent data, *Proteus* species isolated from the urinary tract exhibit increased resistance to β-lactams, often associated with a history of complicated infections [4,45]. Confirmed also in our research, the literature continues to suggest that aminoglycosides and carbapenems remain the most appropriate treatment options for *Proteus* species [45,46].

Species-specific analysis of Gram-negative bacteria in men revealed consistently higher resistance levels than those observed in women, encompassing multiple classes of currently used antibiotics. We might conclude that, in our study, Gram-negative bacteria had an overall resistance rate ratio that increased. According to recently published studies, *E. coli* and *Klebsiella* exhibit increasingly high levels of resistance to antibiotics such as β-lactams, fluoroquinolones, and trimethoprim-sulfamethoxazole, with resistance rates exceeding 30–40% in clinical data [4,30]. Our study also reinforces this positive pattern in resistance rates.

The most viable options, with preserved sensitivity and the most proven therapeutic options when necessary, remain aminoglycosides and carbapenems, which continue to maintain sensitivity against most bacterial species [30,42].

The resistance data reported in this study for Gram-negative bacteria are consistent with epidemiological data showing a global trend of increasing microbial resistance [42].

### 4.4. Gram-Positive Comparison

The most commonly identified Gram-positive bacterium, *Enterococcus*, showed a relatively uniform distribution of resistance and susceptibility rates across all patients, both men and women. As is known, this species shows increased resistance to β-lactams and fluoroquinolones, particularly in patients diagnosed with recurrent UTI or with prior UTI experience (even if unique). This study also demonstrates increased resistance rates to antibiotics such as ampicillin and fluoroquinolones [46,47]. A positive finding identified in both the specialized studies and the present study is that *Enterococcus* maintained relatively high sensitivity rates, with varying responses depending on the patient’s sex, to several classes of antibiotics, including linezolid, fosfomycin, nitrofurantoin, and vancomycin [2,30,42]. These findings demonstrate the continued availability of viable treatment options in clinical practice for urinary tract infections caused by *Enterococcus*.

In a series of previous epidemiological studies that identify this bacterial species as the most common Gram-positive pathogen, it has been demonstrated that it is also the most frequently isolated pathogen among elderly individuals, hospitalized patients, and those requiring indwelling catheterization [30,46]. In addition, current data indicate that the resistance pattern of *Enterococcus* maintains a much more stable resistance profile than that of Gram-negative species, even though it exhibits increased resistance to certain antibiotics, such as fluoroquinolones [42,47,48].

Last but not least, the second most frequently identified Gram-positive species, *Staphylococcus*, showed significant differences between the two study groups regarding resistance rates to levofloxacin and penicillin. Similar data have been reported in the literature that prove increased resistance to fluoroquinolones and penicillin derivatives for *Staphylococcus* isolates identified in urine cultures [4,49]. Nitrofurantoin, linezolid, and vancomycin exhibited consistent susceptibility patterns, consistent with the data. This can suggest that glycopeptides and oxazolidinones maintain stable susceptibility profiles against Gram-positive bacteria [4,47]. The data suggest that even if increased resistance to some commonly used antibiotics is found, viable treatment options still exist for *Staphylococcus*-UTIs [47].

Although Gram-positive uropathogens demonstrated more stable resistance patterns compared to Gram-negative bacteria, their clinical relevance remains important. The preserved susceptibility to linezolid, vancomycin, and fosfomycin observed in our study is consistent with previous reports confirming their effectiveness against *Enterococcus* and *Staphylococcus* species [21,47,48,50,51]. However, the relatively high resistance rates to fluoroquinolones and β-lactams highlight the limited utility of these agents in empirical therapy. Notably, the absence of major sex-related differences suggests that host-related anatomical factors may have a lesser impact on resistance patterns in Gram-positive infections compared to Gram-negative pathogens.

### 4.5. Strengths and Limitations

This study has numerous strengths, primarily due to the fact that it includes nearly 4000 positive urine cultures identified in both the male and female populations, representing one of the largest databases studied in the Romanian population. A second strength is that the investigation was conducted at a high-volume tertiary urology center. In addition to the hospital’s high patient volume and collaboration with other centers, the hospital provides investigations and treatment for the most complex pathologies, including lithiasis, benign urological conditions, functional disorders, and cancer-related pathologies. Thirdly, this study directly compares the results of positive urine cultures identified in the two genders analyzed, which is very rare in specialized studies. In addition to all this, the extended 3-year period enables a comprehensive assessment of microbial resistance patterns observed in the Romanian population.

This study has several limitations. First, only the initial positive urine culture per patient was included, precluding analysis of recurrent infections. Second, the use of six-month intervals rather than a full-year assessment may introduce seasonal bias. The retrospective design represents an inherent limitation, as it relies on previously recorded data, which may be incomplete, inconsistently documented, and subject to selection bias, thereby limiting control over potential confounding factors. In addition, the inclusion of both inpatients and outpatients may have introduced population heterogeneity and restricted the availability of detailed clinical data, particularly for outpatients, limiting the analysis of associated pathologies. Furthermore, as this study was conducted in a tertiary referral center managing complex urological cases, the observed antimicrobial resistance rates may be higher than those in the general population. The exclusion of patients with urinary catheters further limits the generalizability of the findings. In addition, asymptomatic bacteriuria was not specifically evaluated, and lithangiuria was not systematically assessed, potentially leading to an incomplete characterization of the bacteriological spectrum. Another limitation is the absence of multivariate analysis to adjust for potential confounders such as age, comorbidities, or healthcare exposure. This was primarily due to the retrospective design and the incomplete availability of detailed clinical data, particularly for outpatient cases. Finally, the single-center design limits external validity, and caution is warranted when extrapolating these results to broader populations.

## 5. Conclusions

This study demonstrates significant demographic and microbiological differences between male and female patients with urinary tract infections, with males presenting at an older age, a lower prevalence of *Escherichia coli*, and a higher prevalence of *Klebsiella* spp. and *Pseudomonas aeruginosa*.

Statistically significantly higher resistance rates were observed in male patients for several Gram-negative pathogens, particularly to fluoroquinolones, trimethoprim/sulfamethoxazole, and amoxicillin-clavulanic acid. These findings likely reflect the higher burden of complicated infections and increased healthcare exposure in a tertiary-care setting.

Among Gram-negative bacteria, *Klebsiella* spp. exhibited the most concerning resistance profile, including notable resistance to multiple antibiotic classes and carbapenems.

In contrast, Gram-positive bacteria showed relatively stable sensitivity patterns, with preserved activity of fosfomycin, nitrofurantoin, linezolid, and vancomycin, without significant sex-related differences.

These results highlight the importance of sex-specific and local resistance data in guiding empirical therapy, particularly in male patients. Further prospective studies with multivariate analysis are needed to clarify independent risk factors for antimicrobial resistance.

## Figures and Tables

**Table 1:** Age distribution.

	<55 Years	>55 Years	
Female	575	1133	1708
Male	247	2023	2270

**Table 2:** Patients’ setting.

	Females		Males	
	No.	%.	No.	%.
Inpatient	647	37.9	965	42.5
Outpatient	1061	62.1	1305	57.5
Total	1708	100	2270	100

**Table 3:** Bacterial Gram distribution.

		Female		Male	
Group	Gram	Nr.	%.	Nr.	%.
Bacteria	Gram Negative	1369	80.15	1752	77.18
	Gram Positive	339	19.85	518	22.82

**Table 4:** Bacterial prevalence in the analysed groups.

		Female Nr.	Female %.	Male Nr.	Male%.
Gram Negative	*E. coli*	924	54.1	736	32.42
	*Klebsiella*	263	15.4	586	25.81
	*Proteus*	123	7.2	201	8.85
	*Pseudomonas*	59	3.45	229	10.09
Gram Positive	*Enterococcus*	267	15.63	424	18.68
	*Staphylococcus*	72	4.22	94	4.14

**Table 5:** Bacterial distribution—patients’ setting.

		Females		Males	
		Inpatient No./%	Outpatient No./%	Inpatient No./%	Outpatient No./%
Gram Negative	*E. coli*	326/35.3	598/64.7	191/39.5	445/60.5
	*Klebsiella*	101/38.4	162/61.6	275/46.9	311/53.1
	*Proteus*	57/46.3	66/53.7	84/41.8	117/58.2
	*Pseudomonas*	23/39.0	36/61.0	103/45.0	126/55.0
Gram Positive	*Enterococcus*	112/41.9	155/58.1	179/42.2	245/57.8
	*Staphylococcus*	28/38.9	44/61.1	33/35.1	61/64.9
Pearson Chi-Square		8.364		10.077	
*p* Value		0.137		0.073	

**Table 6:** Gram negative (Gram −) Overall resistance rates.

Antibiotic	Gram − R		Gram − S		Gram − R		Gram − S	
	Female				Male			
	Nr.	%.	Nr.	%.	Nr.	%.	Nr.	%.
Amikacin	125	9.31	1218	90.69	222	13.14	1468	86.86
Amoxicillin-Clavulanic Acid	398	30.92	889	69.08	685	49.14	709	50.86
Aztreonam	125	13.89	775	86.11	225	27.47	594	72.53
Trimethoprim/ Sulphametoxazole	141	34.64	266	65.36	417	46.38	482	53.62
Ceftazidime	191	14.13	1161	85.87	429	25.7	1240	74.3
Fosfomycin	3	0.33	903	99.67	21	3.0	678	97.0
Imipenem	44	3.34	1272	96.66	172	15.79	917	84.21
Levofloxacin	425	31.48	925	68.52	705	42.96	936	57.04
Meropenem	41	3.21	1235	96.79	125	15.26	694	84.74
Nitrofurantoin	104	12.35	738	87.65	238	28.17	607	71.83

**Table 7:** Gram positive (Gram +) Overall resistance rates.

Antibiotic	Gram + R		Gram + S		Gram + R		Gram + S	
	Female				Male			
	Nr.	%.	Nr.	%.	Nr.	%.	Nr.	%.
Amikacin	6	9.38	58	90.62	2	9.52	19	90.48
Ampicillin	43	16.6	216	83.4	46	12.2	331	87.8
Trimethoprim/ Sulphametoxazole	15	22.73	51	77.27	31	30.69	70	69.31
Ceftazidime	9	25.0	27	75.0	5	27.78	13	72.22
Fosfomycin	11	4.26	247	95.74	18	4.88	351	95.12
Levofloxacin	128	39.02	200	60.98	234	49.89	235	50.11
Linezolid	13	4.05	308	95.95	16	3.41	453	96.59
Nitrofurantoin	21	6.67	294	93.33	26	5.54	443	94.46
Penicillin	123	38.32	198	61.68	198	42.49	268	57.51
Vancomycin	13	4.96	249	95.04	9	2.25	391	97.75

**Table 8:** *E. coli* resistance and sensitivity rates.

Antibiotic	*E. coli* R		*E. coli* S		*E. coli* R		*E. coli* S		*p* Value
	Female				Male				
	Nr.	%.	Nr.	%.	Nr.	%.	Nr.	%.	
Amikacin	75	8.29	830	91.71	33	4.69	670	95.31	0.239
Amoxicillin-Clavulanic Acid	248	27.19	664	72.81	272	40.18	405	59.82	0.012
Aztreonam	53	8.94	540	91.06	50	16.34	256	83.66	<0.001
Trimethoprim/ Sulphametoxazole	98	32.89	200	67.11	196	44.65	243	55.35	0.001
Ceftazidime	92	10.07	822	89.93	113	16.17	586	83.83	<0.001
Fosfomycin	2	0.23	861	99.77	10	1.53	644	98.47	0.004
Imipenem	4	0.45	891	99.55	5	1.13	438	98.87	0.151
Levofloxacin	281	30.78	632	69.22	296	43.27	388	56.73	0.023
Meropenem	2	0.23	858	99.77	2	0.82	242	99.18	0.178
Nitrofurantoin	38	5.28	682	94.72	49	8.86	504	91.14	0.012

**Table 9:** *Klebsiella* resistance and sensitivity rates.

Antibiotic	*Klebsiella* R		*Klebsiella* S		*Klebsiella* R		*Klebsiella* S		*p* Value
	Female				Male				
	Nr.	%.	Nr.	%.	Nr.	%.	Nr.	%.	
Amikacin	27	10.38	233	89.62	109	19.33	455	80.67	0.069
Amoxicillin-Clavulanic Acid	109	43.25	143	56.75	323	60.6	210	39.4	0.034
Aztreonam	48	27.27	128	72.73	98	37.4	164	62.6	0.027
Trimethoprim/ Sulphametoxazole	23	31.08	51	68.92	151	43.64	195	56.36	0.046
Ceftazidime	62	24.03	196	75.97	204	36.23	359	63.77	<0.001
Imipenem	13	5.24	235	94.76	82	23.1	273	76.9	0.012
Levofloxacin	72	27.91	186	72.09	223	40.18	332	59.82	<0.001
Meropenem	16	6.45	232	93.55	46	18.18	207	81.82	0.014
Nitrofurantoin	61	55.96	48	44.04	180	65.69	94	34.31	0.075

**Table 10:** *Pseudomonas* resistance and sensitivity rates.

Antibiotic	*Pseudomonas* R		*Pseudomonas* S		*Pseudomonas* R		*Pseudomonas* S		*p* Value
	Female				Male				
	Nr.	%.	Nr.	%.	Nr.	%.	Nr.	%.	
Amikacin	18	31.58	39	68.42	70	30.84	157	69.16	0.927
Aztreonam	17	39.53	26	60.47	73	44.24	92	55.76	0.580
Imipenem	22	39.29	34	60.71	73	38.83	115	61.17	0.952
Levofloxacin	31	53.45	27	46.55	105	49.53	107	50.47	0.598
Meropenem	21	36.84	36	63.16	73	34.93	136	65.07	0.790

**Table 11:** *Proteus* resistance and sensitivity rates.

Antibiotic	*Proteus* R		*Proteus* S		*Proteus* R		*Proteus* S		*p* Value
	Female				Male				
	Nr.	%.	Nr.	%.	Nr.	%.	Nr.	%.	
Amikacin	5	4.13	116	95.87	10	5.1	186	94.9	0.987
Amoxicillin-Clavulanic Acid	32	29.09	78	70.91	83	47.43	92	52.57	<0.001
Aztreonam	7	7.95	81	92.05	4	4.65	82	95.35	0.373
Trimethoprim/ Sulphametoxazole	20	58.82	14	41.18	69	61.61	43	38.39	0.773
Ceftazidime	11	8.94	112	91.06	34	17.99	155	82.01	0.026
Imipenem	5	4.27	112	95.73	12	11.65	91	88.35	0.041
Levofloxacin	41	33.88	80	66.12	81	42.63	109	57.37	0.124
Meropenem	2	1.8	109	98.2	4	3.54	109	96.46	0.423

**Table 12:** *Enterococcus* resistance and sensitivity rates.

Antibiotic	*Enterococcus* R		*Enterococcus* S		*Enterococcus* R		*Enterococcus* S		*p* Value
	Female				Male				
	Nr.	%.	Nr.	%.	Nr.	%.	Nr.	%.	
Ampicillin	41	16.02	215	83.98	46	12.23	330	87.77	0.176
Fosfomycin	11	4.3	245	95.7	18	4.92	348	95.08	0.718
Levofloxacin	112	42.75	150	57.25	187	48.83	196	51.17	0.128
Linezolid	9	3.54	245	96.46	14	3.67	367	96.33	0.931
Nitrofurantoin	17	6.61	240	93.39	22	5.68	365	94.32	0.628
Penicillin	85	33.6	168	66.4	126	33.33	252	66.67	0.945
Vancomycin	12	4.69	244	95.31	9	2.26	390	97.74	0.085

**Table 13:** *Staphylococcus* resistance and sensitivity rates.

Antibiotic	*Staphylococcus* R		*Staphylococcus* S		*Staphylococcus* R		*Staphylococcus* S		*p* Value
	Female				Male				
	Nr.	%.	Nr.	%.	Nr.	%.	Nr.	%.	
Trimethoprim/ Sulphametoxazole	14	21.54	51	78.46	30	34.88	56	65.12	0.075
Ceftazidime	9	28.12	23	71.88	1	100.0	0	0.0	0.146
Levofloxacin	16	24.24	50	75.76	47	54.65	39	45.35	<0.001
Linezolid	4	5.97	63	94.03	2	2.27	86	97.73	0.240
Nitrofurantoin	4	6.9	54	93.1	4	4.88	78	95.12	0.617
Penicillin	38	55.88	30	44.12	72	81.82	16	18.18	<0.001

## Data Availability

Data supporting the reported results are available from the authors.
